# Deprescribing of proton pump inhibitors in older patients: A cost-effectiveness analysis

**DOI:** 10.1371/journal.pone.0311658

**Published:** 2024-10-07

**Authors:** Mingxi Xie, Joyce H. S. You

**Affiliations:** School of Pharmacy, Faculty of Medicine, The Chinese University of Hong Kong, Hong Kong SAR, China; Birjand University of Medical Sciences, ISLAMIC REPUBLIC OF IRAN

## Abstract

Over-prescribing of proton-pump inhibitors (PPIs) is widely observed in older patients. Clinical findings have showed that deprescribing service significantly decreased inappropriate PPIs utilization. We aimed to examine the cost-effectiveness of PPI deprescribing service from the perspective of Hong Kong public healthcare provider. A decision-analytic model was constructed to examine the clinical and economic outcomes of PPI deprescribing service (deprescribing group) and usual care (UC group) in a hypothetical cohort of older PPI-users aged ≥65 years in the ambulatory care setting. The model inputs were retrieved from literature and public data. The model time-frame was one-year. Base-case analysis and sensitivity analysis were performed. Primary model outcomes were direct medical cost and quality-adjusted life-years (QALYs) loss. In base-case analysis, the deprescribing service (versus UC) reduced total direct medical cost by USD235 and saved 0.0249 QALY per PPI user evaluated. The base-case results were robust to variation of all model inputs in one-way sensitivity analysis. In probabilistic sensitivity analysis, the deprescribing group was accepted as cost-effective (versus the UC group) in 100% of the 10,000 Monte Carlo simulations. In conclusion, the PPI deprescribing service saved QALYs and reduced total direct medical cost in older PPIs users, and showed a high probability to be accepted as the cost-effective option from the perspective of public healthcare provider in Hong Kong.

## Introduction

Proton-pump inhibitors (PPIs), the primary treatment for gastroesophageal reflux disease (GERD), have become the most popular prescription medication. It is estimated that PPIs utilization increased about 10.4-fold from 2004 to 2013 in Southwest China [1]. Over-prescription of PPIs was observed in 73.9% of older patients and PPIs use with non-evidence-based indications was found in approximately 60% of community-dwelling older patients [2]. In addition to the financial burden to healthcare system, PPIs was associated with increased risk of adverse events including pneumonia, Clostridium difficile infection (CDI) and hypomagnesemia [3].

A clinical practice guideline recommends deprescribing of PPIs in adults who have administered PPIs for at least 4 weeks to treat mild to moderate gastroesophageal reflux diseases, heartburn or esophagitis, and whose symptoms have been completely resolved [4]. Clinical findings have showed that the deprescribing service significantly decreased PPIs utilization and medication expenditure in ambulatory care settings, and resulted in high rate of PPI deprescribing completion (80.0%) [5, 6]. However, the provision of deprescribing service requires manpower costs, and deprescribing may cause GERD rebound [7]. To facilitate the clinical and administrative decision-makers on resource allocation for the PPI-deprescribing service, we aimed to analyse the cost-effectiveness of PPI deprescribing in older patients from the perspective of public healthcare provider in Hong Kong.

## Materials and methods

### Model design

**Fig 1:**
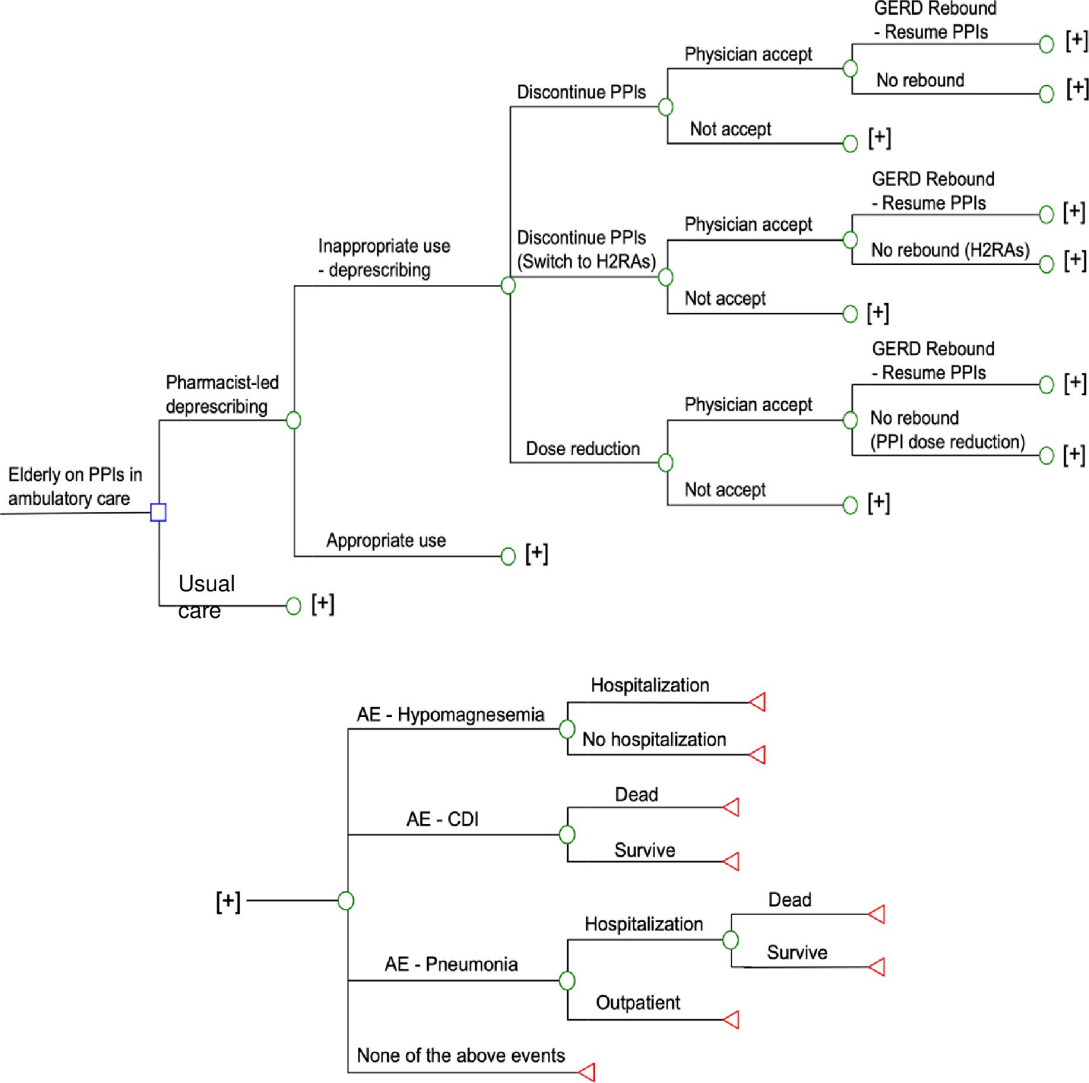
Simplified decision-analytical model of proton pump inhibitor (PPI) deprescribing service versus usual care in older PPIs users. AE: adverse event; GERD: gastroesophageal reflux disease; H2RAs: Histamine-2 receptor antagonists; PPIs: proton-pump inhibitors.

A decision-analytical model (**Fig 1**) was constructed to examine the clinical and economic outcomes of PPI deprescribing (deprescribing group) and usual care (UC group) in a hypothetical cohort of older PPI-users aged ≥65 years in the ambulatory care setting. Decision tree is a form of decision-analytical model, and hypothetical patients proceed through health states based on probability inputs of the model. A recent retrospective study (n = 402) reported that the mean onset of rebound symptoms was less than 20 days after PPI discontinuation [8]. A one-year time horizon was therefore applied in the present model to allow enough time for capturing the impact of deprescribing PPIs. Primary model outcomes were direct medical cost and quality-adjusted life-years (QALYs) loss.

Deprescribing is a planned process to discontinue or reduce doses of medications which do not provide benefits or cause harm to patients, under the supervision of healthcare professionals [4]. In the deprescribing group, a pharmacist-led PPI deprescribing service evaluated the appropriateness of PPI regimens and made one of the following recommendations to the prescriber for the inappropriate cases: PPI discontinuation, PPI dose reduction, and switching PPI to histamine-2 receptor antagonists (H2RAs) [4]. The prescriber might (or might not) accept the deprescribing recommendations. If GERD rebound occurred after deprescribing PPI, the PPI regimen would be reinstated [7]. In the UC group, no deprescribing service was provided and patients continued the PPI regimens.

In both study groups, all patients (PPI continued, dose-reduced, discontinued, or switched to H2RA) might (or might not) experience adverse events: Pneumonia, CDI, or hypomagnesemia [3]. Patients with hypomagnesemia might receive inpatient treatment. Low serum magnesium was associated with cardiovascular symptoms, but the direct causal relationship between hypomagnesemia and cardiac death was not supported [9]. Mortality associated with hypomagnesemia therefore was not included in the model. Patients with CDI were hospitalized and CDI-related deaths might occur. Patients with pneumonia might receive outpatient or inpatient treatment, and the hospitalized patients might survive or die.

### Clinical inputs

A literature search was conducted on PubMed and Google Scholar over the period 1995–2023. Search terms including (but not limited to) “proton pump inhibitors”, “gastroesophageal reflux disease”, “older adults”, “elderly”, “deprescribing”, “inappropriate use”, “overutilization”, “rebound symptoms”, “pneumonia”, “Clostridium difficile infection”, and “hypomagnesemia” were used as keywords and (if applicable) Medical Subject Headings (MeSH) terms. The selection criteria of studies for clinical model inputs of PPI-related outcomes were: (1) English-written reports; (2) patients aged ≥65 years using PPIs; and (3) one or more of the followings were reported: Incidence rate of inappropriate PPI use; prescribers’ acceptance to alternatives for replacing PPI therapy; symptomatic rebound rates after PPI deprescribing; adverse event (hypomagnesemia, pneumonia, Clostridium difficile infection) rates in PPI and non-PPI users. To estimate the clinical outcomes of adverse events (hypomagnesemia, pneumonia, Clostridium difficile infection), outcomes studies written in English were retrieved in literature using search terms such as “hypomagnesemia”, “pneumonia”, “Clostridium difficile infection”, “elderly”, “outcomes” as keywords and/or MeSH terms. Meta-analysis and randomized controlled trials were the preferred study types during the literature search. When an event/incidence rate was reported in multiple studies, the weighted average was used as the base-case value, and the highest and lowest values were considered in the sensitivity analysis.

The proportion of inappropriate PPI uses (78.52%; range 73.93%–84.05%) was approximated from findings of two cohort studies (total older patients on PPIs n = 512) on PPI use evaluation [10, 11]. The distribution of PPI deprescribing interventions were estimated from findings of an observational study of 29,694 pharmacist interventions on PPIs: PPI discontinuation (32.79%; range 29.51%–36.07%), PPI dose reduction (22.25%; range 20.03%–24.48%) and drug switch (44.96%; range 40.46%–49.45%). The prescriber acceptance rates on recommendations of PPI discontinuation (73.1%; range 65.79%–80.41%), PPI dose reduction (70.3%; range 63.27%–77.33%) and drug switch (88.0%; range: 79.20%–96.80%) were also reported by this study and adopted in the present model [12]. The occurrence of GERD rebound (78.67%; range: 70.80%–86.53%) after PPI discontinuation in long-term PPIs users (n = 97) was retrieved from a double-blind, placebo-controlled trial [13]. The GERD rebound after PPI dose reduction (20.51%; range 18.46%–22.56%) was approximated from the findings of a prospective study (n = 117) on PPI dose step-down in patients in remission of acid regurgitation [14]. The GERD rebound after drug switch to H2RAs (42.58%; range 38.32%–46.83%) was the pooled average of GERD treatment failure rate in H2RAs users [15–19].

The yearly event rates of adverse events in PPIs users were estimated using the yearly rate of an adverse event in non-PPI users and the corresponding odd ratios (OR) in PPIs users. The yearly event rate of hypomagnesemia in non-PPI users (6.89%; range 6.20%–7.58%) and the OR for PPIs users (1.775; 95%CI 1.077–2.924) were reported from a meta-analysis including nine studies with 115,455 patients [20]. The yearly event rate of pneumonia in non-PPI users (5.37%; range 4.83%–5.91%) was retrieved from data of a meta-analysis (8 studies) [21]. The OR of pneumonia for PPI users (1.490; 95%CI 1.16–1.92) was reported by a meta-analysis (26 studies), and the OR for low-dose PPIs users (1.173; 95%CI 1.110–1.239) was obtained from a sub-group meta-analysis (9 studies) [22, 23]. The CDI yearly event rate in non-PPI users (4.08%; range: 3.67%–4.48%) and the OR for PPIs users (2.15; 95%CI 1.81–2.55) were adopted from the findings of a meta-analysis (25 case-control and 5 cohort studies) [24]. The OR of CDI for low-dose PPIs users (1.28; 95%CI 0.94–1.74) was approximated from the findings of a hospital cohort study (n = 157,693) on risk of CDI associated with PPIs use [25].

The hospitalization rate for hypomagnesemia (0.00131%; range 0.00118%–0.00144%) was estimated from a 10-year population-based case-control study of PPIs use and hospitalization with hypomagnesemia in older individuals [26]. The hospitalization rate for pneumonia (33.93%; range 30.54–37.32%) was retrieved from findings of a prospective study on hospitalization decision of ambulatory patients aged ≥65 years with pneumonia [27]. The in-hospital mortality rate of CDI in the older patients (8.8%; range:7.92%–9.68%) was reported by a retrospective analysis of 2.3 million community hospital discharges in the US [28]. The mortality rate of pneumonia among hospitalized older patients (11.0%; range: 9.9%–12.1%) was reported in a US national health care survey (n = 76857) [29].

### Utility inputs

The QALY loss of each hypothetic patient was estimated using the age-specific health utility, health state-specific disutility, and time spent in the health state. The utility value for individuals aged ≥65 years (0.81; range 0.729–0.891) was adopted from the preference-based score in the US [30]. The disutility values for GERD symptoms with medications (0.0486; range 0–0.405) and without medications (0.0737; range 0–0.567) were retrieved from an observational study of 222 GERD patients [31]. The disutility value of patients hospitalized for hypomagnesemia (0.2430; range 0.1130–0.3730) was estimated from the EQ-5D measures of arrhythmia reported by a national cross-sectional survey, as arrhythmia was the main severe symptom of hypomagnesemia [32]. The disutility scores of pneumonia treated by hospitalization (0.5597; range 0.5022–0.6164) and treated by ambulatory care (0.3013; range 0.2649–0.3370) were estimated from outcomes of a cross-sectional analysis targeting pneumococcal diseases [33]. The disutility score of CDI (0.3985; range 0.3587–0.4384) was approximated from findings of prospective study on utility decrement associated with CDI [34]. The length of hospitalization for hypomagnesemia (6 days; range 4–9), pneumonia (9.8 days; range 9.3–10) and CDI (8 days; range 5–14) were retrieved from relevant health outcome studies [28, 35, 36]. The convalescence period of pneumonia (31 days; range 13–55) was estimated from the findings of a population-based epidemiological study (n = 241) [37]. The QALY loss for death (associated with pneumonia or CDI) was calculated by the age-specific utility and remaining life expectancy. The age-specific remaining life expectancy was obtained from the life expectancy table reported by the Census and Statistics Department of Hong Kong [38]. In this model, the base-case value of patient age (80 years; range 65–88 years) was adopted from a prospective study on PPIs overutilization in older patients [11]. The QALY loss due to mortality was discounted to the current year at 3% annual rate.

### Cost inputs

Costs analysis was conducted from the perspective of public healthcare provider in Hong Kong. Costs of public healthcare services were estimated by the unit cost and utilization. Hospitalization daily cost and cost per clinic visit were retrieved from the Hospital Authority, the public healthcare provider in Hong Kong [39]. The number of clinic visits required for pneumonia (3, range 2–4) was estimated from a health economic study on community-acquired pneumonia [40]. The length of hospitalization of adverse events were described above in the “Utility inputs” section. Monthly medication costs of standard doses of PPIs, low doses of PPIs and standard doses of H2RAs were drug acquisition costs adjusted to current year [41]. The cost of deprescribing PPI service was estimated by the salary of pharmacist and the time spent (34 minutes; range 28–40) on each case, reported by a pharmacist-led deprescribing pilot program [42, 43]. The present model timeframe was one year, and discounting was therefore not applied to model cost outputs. All model parameters were shown in **Table 1**.

**Table 1:** Model inputs.

Variables	Base-case value	Range for sensitivity analysis	Distribution	reference
*Clinical Inputs*				
Inappropriate PPI use	0.7852	0.7393–0.8405	Beta	[10, 11]
Deprescribing service recommendations on inappropriate PPI use				
PPIs discontinuation	0.3279	0.2951–0.3607	Beta	[12]
PPIs dose reduction	0.2225	0.2003–0.2448	Beta	[12]
Switch to H2RAs	0.4496	0.4046–0.4945	Beta	[12]
Prescribers’ acceptance to recommendations				
PPIs discontinuation	0.7310	0.6579–0.8041	Beta	[12]
PPIs dose reduction	0.7030	0.6327–0.7733	Beta	[12]
Switch to H2RAs	0.8800	0.7920–0.9680	Beta	[12]
Rebound rates				
PPIs discontinuation	0.7867	0.7080–0.8653	Beta	[13]
PPIs dose reduction	0.2051	0.1846–0.2256	Beta	[14]
Switch to H2RAs	0.4258	0.3832–0.4683	Beta	[15–19]
Yearly event rate in non-PPI users				
Hypomagnesemia	0.0689	0.0620–0.0758	Beta	[20]
Pneumonia	0.0537	0.0483–0.0591	Beta	[21]
Clostridium difficile infection	0.0408	0.0367–0.0448	Beta	[24]
Odd ratios for PPI users				
Hypomagnesemia	1.775	1.077–2.924	Triangular	[30]
Pneumonia	1.490	1.160–1.920	Triangular	[22]
Clostridium difficile infection	2.150	1.810–2.550	Triangular	[24]
Odd ratios for PPI users with low dose				
Hypomagnesemia	1.775	1.077–2.924	Triangular	[20]
Pneumonia	1.173	1.110–1.239	Triangular	[23]
Clostridium difficile infection	1.280	0.940–1.740	Triangular	[25]
Hospitalization rates				
Hypomagnesemia	0.0000131	0.0000118–0.0000144	Beta	[26]
Pneumonia	0.3393	0.3054–0.3732	Beta	[27]
In-hospital mortality rates				
Clostridium difficile infection	0.0880	0.0792–0.0968	Beta	[28]
Pneumonia	0.1100	0.0990–0.1210	Beta	[29]
*Utility inputs*				
Age (years)	80	65–88	Triangular	[11]
Health Utility for older persons aged > 65	0.810	0.729–0.891	Triangular	[30]
Disutility values for symptomatic relapse				
On medications	0.0486	0–0.405	Triangular	[31]
Off medications	0.0737	0–0.567	Triangular	[31]
Disutility values for adverse events				
Hospitalization for hypomagnesemia	0.2430	0.1130–0.3730	Triangular	[32]
Hospitalized pneumonia	0.5597	0.5022–0.6164	Triangular	[33]
Ambulatory pneumonia	0.3013	0.2649–0.3370	Triangular	[33]
Clostridium difficile infection	0.3985	0.3587–0.4384	Triangular	[34]
Length of hospital stay (days)				
Hypomagnesemia	6	4–9	Triangular	[36]
Pneumonia	9.8	9.3–10	Triangular	[35]
Clostridium difficile infection	8	5–14	Triangular	[28]
Convalescence period for pneumonia (days)	31	13–55	Triangular	[37]
Length for outpatient pneumonia (days)	31	13–55	Triangular	[37]
*Cost Inputs*				
Direct monthly medication costs (USD)				
Standard dose of PPIs	54	47–65	Gamma	[41]
Low dose of PPIs	42	38–47	Gamma	[41]
Standard dose of H2RA	6	1.7–10	Gamma	[41]
Public healthcare services fees				
Hospitalization cost per day (USD)	654	-		[39]
Clinic cost per visit (USD)	57	-		[39]
Clinic visits for pneumonia	3	2–4	Triangular	[40]
Deprescribing service				
Pharmacist monthly salary (USD)	10,918	7,358–14,478	Gamma	[42]
Pharmacist-time spent on deprescribing (minutes per case)	34	28–40	Triangular	[43]

### Cost-effective analysis and sensitivity analyses

TreeAge Pro 2023 (TreeAge Software Inc, Williamstown, MA, USA) and Excel 365 (Microsoft Corporation, Redmond, WA, USA) were used to conduct the cost-effectiveness and sensitivity analyses. The deprescribing group was cost-effective if it (1) saved QALY at lower cost (dominant), or (2) saved QALY at higher cost and the incremental cost per QALY gained (ICER = Incremental Cost/ QALY saved) was below the willingness-to-pay (WTP) threshold. The World Health Organization suggested that a strategy is highly cost-effective if the ICER is less than 1× gross domestic product (GDP) per capita [44]. The GDP per capita of Hong Kong in 2022 (USD49,023; USD1 = HKD7.8) was therefore adopted as the WTP threshold [45].

The range for sensitivity analysis of each model input was formed by the range, 95% confidence interval (if available), or ± 10% of the base-case value. One-way sensitivity analysis was conducted by varying each model parameter over the range for sensitivity analysis. Monte Carlo simulations were used for probabilistic sensitivity analysis. The cost and QALY loss of both study groups were simulated 10,000 times by simultaneously drawing all model parameters from the parameter-specific distribution specified in **Table 1**. The incremental cost and QALY saved by the deprescribing group versus UC group in the Monte Carlo simulations were presented in a scatter plot.

## Results and discussion

### Base-case analysis results

The expected cost and QALY loss of each study group are shown in **Table 2**. Comparing with the UC group, the deprescribing group saved 0.0249 QALYs and reduced cost by USD235 per PPI user. The deprescribing group was dominant and therefore preferred as the cost-effective option from the perspective of public healthcare provider in Hong Kong.

**Table 2:** Base-case results.

Strategy	Cost (USD)	Incremental Cost (USD)	QALY Loss	QALY saved	Incremental cost-effectiveness ratio (ICER)
Usual care	1,296	-	0.2397	-	-
PPI Deprescribing	1,061	-235	0.2148	0.0249	-9,438 (Dominant)

PPI Deprescribing saved QALY at lower cost (dominant) and was accepted as cost-effective.

### Sensitivity analyses results

**Fig 2:**
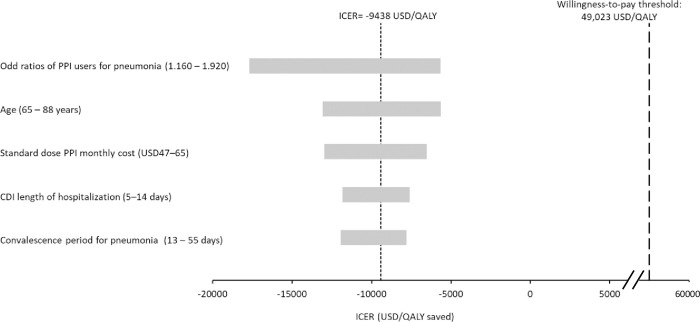
Tornado diagram of top 5 influential factors in one-way sensitivity analysis on incremental cost-effectiveness ratio (ICER) of the proton pump inhibitors deprescribing service versus usual care.

One-way sensitivity analysis was performed on all model inputs, and no threshold value was found. The deprescribing group remained QALY-saving at lower total cost when comparing to the UC group throughout one-way sensitivity analysis of all model inputs. The top five influential model parameters on the ICER of deprescribing group are showed in a tornado diagram (**Fig 2**).

**Fig 3:**
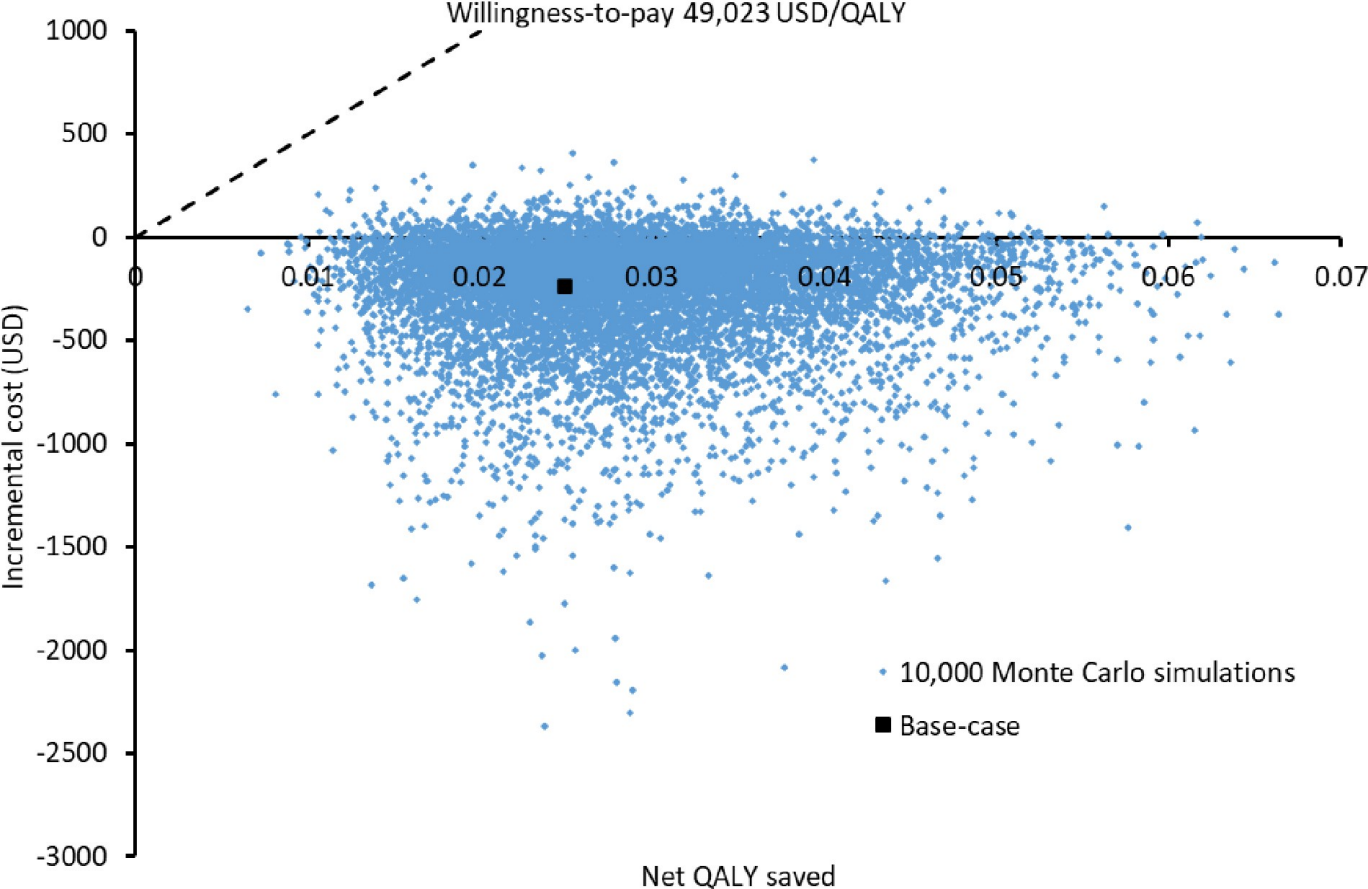
Scatter plot of the incremental cost against QALY gained by the proton pump inhibitors deprescribing service versus usual care in 10,000 Monte Carlo simulations.

Probabilistic sensitivity analysis was performed by conducting 10,000 Monte Carlo simulations on direct medical costs and QALY loss for each study group. When comparing to the UC group, QALYs and medical costs saved by the deprescribing group were 0.02891 QALYs (95%CI 0.02874–0.02908; p< 0.001) and USD248 (95%CI 243–254; p<0.001), respectively. The incremental costs and QALYs saved by the deprescribing group versus UC group were shown in the scatter plot (**Fig 3**). Comparing with the UC group, the deprescribing group gained QALYs and saved cost in 100% and 91.5% of 10,000 simulations, respectively. The probability of the deprescribing group to be cost-effective was 100%.

## Discussion

The present cost-effectiveness analysis examined the direct medical costs and QALY saved by the PPI deprescribing service in older PPIs users. The base-case results found the deprescribing service to save cost (by USD235 per PPI user evaluated) and QALYs (by 0.0249 QALYs per PPI user evaluated) from the perspective of public healthcare provider in Hong Kong. The robustness of base-case findings was further demonstrated by one-way and probabilistic sensitivity analyses that the direct costs and QALY loss of the deprescribing group remained lower than those of the UC group.

The cost-saving and QALY gained generated by the deprescribing service were driven by the reduced cases of adverse events associated with PPIs use in older patients. As showed by the top five influential factors on the ICER of deprescribing group in the one-way sensitivity analysis, the economic and clinical parameters of PPI-associated pneumonia and CDI were the drivers of the total cost and QALY loss. Lowering the cases of the major adverse events consequently reduced the direct medical costs and QALY loss. The cost avoidance by reducing adverse events outweighed the cost of deprescribing service, and therefore generated cost-saving.

Deprescribing services led by pharmacists on potentially inappropriate medications in older adults were previously examined, and the effectiveness were measured as the change of hospitalization rate and mortality. A systematic review including nine studies reported that pharmacist-led deprescribing for older adults in ambulatory care settings was effective in reducing the use of inappropriate medications, resulting in reduction in hospitalization and mortality [46]. A cost-utility analysis found that it was potentially cost-saving (by €170.46) with QALY gained (by 0.003 QALYs) to discontinue PPIs in older patients who extended PPIs use after the cessation of non-steroidal anti-inflammatory drugs (NSAID) and low doses of aspirin [47]. Apart from PPIs, the benefits of pharmacist-led deprescribing of other medications have been evaluated. Sanyal et al. reported deprescribing of NSAID by pharmacists in community-dwelling older people was cost-saving (CAD1008.61) and effective (0.11 QALYs gained) when compared to usual care [48]. Furthermore, a cost-utility analysis conducted in Canada demonstrated that pharmacist-led deprescribing of sedatives in community settings was a cost-effective strategy with cost reduction of CAD1392.05 and 0.0769 QALYs gained [49]. The prior cost-effectiveness findings are consistent with results of present study that the deprescribing is a cost-effective service.

Prolonged PPI usage increases risk of adverse outcomes, such as pneumonia, CDI and hypomagnesemia [3]. Despite the successful deprescribing reported in clinical studies [5, 6], the occurrence of symptomatic rebound [7, 8] and uncertainty in cost-effectiveness of PPI deprescribing are hurdles for Hong Kong public health policy makers and budget holders to invest resources to the deprescribing intervention. The present cost-effectiveness study considered the key elements in PPI deprescribing service implementation: Incidence of inappropriate PPI usage, types of deprescribing (discontinuation, dose reduction, change of therapeutic agent) and corresponding acceptance rate and rebound rate, risks of major adverse events associated with PPI users, direct costs of the deprescribing service, and treatment costs and health-related utility values of major adverse events and rebound. Our study provided comprehensive cost-effectiveness evidence to public health policy makers and budget holders to facilitate the informed decision-making process on PPI deprescribing intervention for elderly patients in Hong Kong.

Despite clinical effectiveness findings are generalizable across countries in general, the health economic findings of effective health services are country/region specific. It is therefore important to generate health system- and region-specific cost-effectiveness data to inform the decision-maker on deprescribing services. The decision-analytic model developed in the present study, including the key clinical, disutility and cost inputs for PPI describing, is readily to be adopted in another setting (of different prevalence of inappropriate PPIs use and resource utilization) by using region-specific parameters to inform the model. The model-generated outcomes are also readily to be update with new epidemiology data (when available) to continuously inform the clinicians and healthcare administrators on the cost-effective use of PPI deprescribing service.

The strength of study included translating clinical findings (including epidemiology of inappropriate PPI usage, risk of adverse events associated with PPIs, deprescribing rates of PPI deprescribing intervention, rebound rate after deprescribing) into direct medical costs and QALY. The study provided health economic evidence on the cost-effectiveness of PPI deprescribing by considering both the benefits (avoidance of PPI-associated adverse events) and disadvantages (rebound of symptoms) of the intervention. The weakness of study included limitations associated with uncertainties of model inputs. Majority of model clinical inputs were retrieved from oversea studies, and might affect the generalizability of model results to patients in Hong Kong. Rigorous sensitivity analyses were therefore performed to examine the impact of all model input uncertainties on the robustness of base-case results. The model simplified the adverse events associated with long-term PPIs use to those key events with clinical symptoms (hypomagnesemia, CDI and pneumonia). The simplified model did not capture some undesired outcomes of inappropriate PPIs use, such as changes in gut microbiome [3]. The present analysis was conducted on direct medical costs, and indirect costs including productivity loss were not considered. The present findings might therefore underestimate the total cost saving and QALY gained by the deprescribing intervention.

## Conclusions

In conclusion, the PPI deprescribing service saved QALYs and reduced total direct medical cost in older PPIs users, and showed a high probability to be accepted as the cost-effective option from the perspective of public healthcare provider in Hong Kong.
